# Tetraspanin CD82 is necessary for muscle stem cell activation and supports dystrophic muscle function

**DOI:** 10.1186/s13395-020-00252-3

**Published:** 2020-11-27

**Authors:** Arielle Hall, Tatiana Fontelonga, Alec Wright, Katlynn Bugda Gwilt, Jeffrey Widrick, Alessandra Pasut, Francesco Villa, Cynthia K. Miranti, Devin Gibbs, Evan Jiang, Hui Meng, Michael W. Lawlor, Emanuela Gussoni

**Affiliations:** 1 Division of Genetics and Genomics, Boston Children’s Hospital, Boston, MA 02115 USA; 2 Division of Gastroenterology, Hepatology and Nutrition, Boston Children’s Hospital, Boston, MA 02115 USA; 3 Laboratory of Angiogenesis and Vascular metabolism, Center for Cancer Biology, VIB and KU Leuven, 3000 Leuven, Belgium; 4 F.M. Kirby Neurobiology Center, Boston Children’s Hospital, Harvard Medical School, Boston, MA USA; 5 Department of Cellular and Molecular Medicine, University of Arizona College of Medicine, Tucson, AZ 85724 USA; 6 Molecular Biology Institute, UCLA, Los Angeles, CA 90095 USA; 7 The University of Pennsylvania, College of Arts and Sciences, Philadelphia, PA 19104 USA; 8 Department of Pathology and Laboratory Medicine and Neuroscience Research Center, Medical College of Wisconsin, Milwaukee, WI 53226 USA; 9 The Stem Cell Program at Boston Children’s Hospital, Boston, MA 02115 USA

**Keywords:** Tetraspanin, Stem cells, Muscular dystrophy, Regeneration, mTOR, AKT

## Abstract

**Background:**

Tetraspanins are a family of proteins known to assemble protein complexes at the cell membrane. They are thought to play diverse cellular functions in tissues by modifying protein-binding partners, thus bringing complexity and diversity in their regulatory networks. Previously, we identified the tetraspanin KAI/CD82 as a prospective marker for human muscle stem cells. CD82 expression appeared decreased in human Duchenne muscular dystrophy (DMD) muscle, suggesting a functional link to muscular dystrophy, yet whether this decrease is a consequence of dystrophic pathology or a compensatory mechanism in an attempt to rescue muscle from degeneration is currently unknown.

**Methods:**

We studied the consequences of loss of CD82 expression in normal and dystrophic skeletal muscle and examined the dysregulation of downstream functions in mice aged up to 1 year.

**Results:**

Expression of CD82 is important to sustain satellite cell activation, as in its absence there is decreased cell proliferation and less efficient repair of injured muscle. Loss of CD82 in dystrophic muscle leads to a worsened phenotype compared to control dystrophic mice, with decreased pulmonary function, myofiber size, and muscle strength. Mechanistically, decreased myofiber size in CD82^−/−^ dystrophic mice is not due to altered PTEN/AKT signaling, although increased phosphorylation of mTOR at Ser2448 was observed.

**Conclusion:**

Basal CD82 expression is important to dystrophic muscle, as its loss leads to significantly weakened myofibers and impaired muscle function, accompanied by decreased satellite cell activity that is unable to protect and repair myofiber damage.

**Supplementary Information:**

The online version contains supplementary material available at 10.1186/s13395-020-00252-3.

## Background

The tetraspanins are a class of transmembrane proteins known to regulate the assembly of protein complexes at the cell membrane [1–4]. The tetraspanin-associated proteins are very diverse and change greatly depending on cell type, cellular state, and tissue conditions, such as normal or cancer cells [5]. Thus, the tetraspanin web represents a very dynamic class of proteins that play a role in a number of regulatory networks. Importantly, tetraspanins have been shown to signal through other protein families, such as integrins, making this an extremely broad network with tremendously varied functional translational mechanisms. One tetraspanin, CD82, was first discovered as a potential anti-metastatic marker for prostate cancer [6–8]. It is known to have pro-adhesive properties, and its expression in cancer cells is often linked to good prognostic outcome. CD82 was named as C33, 4F9, IA4, and R2 [9–11] prior to its classification within the clusters of differentiation nomenclature [12]. CD82 has a typical tetraspanin structure, with intracellular N- and C-termini and two extracellular loops, a small (EC1) and a large (EC2) loop [11]. Glycosylation sites are present on the EC2, accounting for additional protein variability, while the transmembrane helix 3 (TM3) and 4 (TM4) domains contain polar residues thought to mediate intramolecular interactions [13]. CD82 has also been shown to interact with various integrin complexes, resulting in the mediation of cellular adhesion to the extracellular matrix by fibronectin and laminin binding [14–16]. While CD82 expression is fairly ubiquitous, there is variation in the transcripts encoded with some isoforms lacking the C-terminus or transcript variants lacking specific exons [17]. While these transcriptional variants are known, their specific function has not been elucidated.

Our previous study reported expression of CD82 in both quiescent and activated human satellite cells, with cells positive for CD82 exhibiting strong myogenic activity both in vitro and in vivo. Recent single cell RNA seq studies have also confirmed robust CD82 expression in mouse satellite cells [18, 19]. In hematopoietic stem cells, CD82 expression is typically indicative of quiescence; thus, CD82 loss of function leads to over-proliferation of cells [20, 21]. In contrast, knock-down of CD82 in myogenic cells derived from induced pluripotent stem cells (iPSCs) reduces myoblast proliferation and impairs cell activation [14, 22]. In human myoblasts, CD82 was shown to associate with α7 integrin and α-sarcoglycan, suggesting it might have a role in linking α7β1 integrin with the sarcoglycan complex [14]. Furthermore, CD82 expression is also decreased in myogenic cell lines and tissue from DMD patients compared to normal controls, while it is increased in regenerating fibers [14]. Whether decreased expression of CD82 in DMD muscle is downstream of the degenerative progressive dystrophic pathology or is part of a compensatory mechanism meant to slow muscle degeneration is currently unclear.

In the present study, we determined the consequences of loss of CD82 function in both normal (CD82^−/−^) and dystrophic (CD82^−/−^:*mdx*^*5cv*^) mouse skeletal muscle. CD82^−/−^ muscles show impaired regeneration following acute injury. While the number of Pax7+ satellite cells CD82^−/−^ mice is not different from WT control, satellite cells from CD82^−/−^ mice exhibit impaired and/or delayed activation and decreased proliferation. We also show that loss of CD82 expression in dystrophic muscle (CD82^−/−^:*mdx*^*5cv*^) leads to a worsened phenotype compared to control *mdx*^*5cv*^ mice. Muscle strength and resistance to mechanical strain are significantly decreased, accompanied by myofiber size being significantly smaller in CD82^−/−^:*mdx*^*5cv*^ mice. Mechanistically, smaller myofibers in CD82^−/−^:*mdx*^*5cv*^ mice are not due to decreased pAKT signaling at neither Ser 473, nor Thr 308, nor to increased PTEN expression. In contrast, mTOR phosphorylation at Ser 2448 was increased in CD82^−/−^:*mdx*^*5cv*^ compared to *mdx*^*5cv*^ mice. In summary, basal CD82 expression is important to dystrophic muscle, as its loss leads to significantly weakened myofibers and impaired muscle function, accompanied by decreased muscle stem cell activity that is unable to protect and repair myofiber damage.

## Methods

### Animal husbandry

Six-week-old male and female wild-type C57BL/6 mice were ordered from the Jackson Laboratories (Bar Harbor, ME) and paired to establish an in-house colony. Mdx^*5cv*^ dystrophic males and females were bred as previously described [23, 24]. Breeding pairs of previously reported CD82 knockout mice (CD82^−/−^) (C57BL/6 background) [25] were obtained from Dr. Cindy Miranti, following approval by the National Cancer Institute Animal Resource Program. CD82^−/−^ mice were bred into *mdx*^*5cv*^ mice (C57BL/6 J background) to generate CD82:dystrophin double-null mice (CD82^−/−^:*mdx*^*5cv*^). Because the dystrophin gene is on the X-chromosome, female mice homozygote for both CD82 and dystrophin allele mutations were generated, while males are homozygotes for the CD82 mutation and hemizygotes for the dystrophin mutation. Male and female mice were analyzed in these studies and cohorts were matched for both age and sex in all comparisons. For each experiment, cohorts of 6-10 mice were used to ensure sufficient statistical power. Mice were bred and euthanized by CO_2_ asphyxiation, followed by cervical dislocation. Mice of all genotypes were analyzed at 2 and 4 months of age, as well as when they reached the age of 1 year. All experiments involving vertebrate animals were approved and conducted in accordance with IACUC Institutional Regulations at Boston Children’s Hospital, Boston, MA.

### Genotyping

Upon weaning at 21 days, CD82^−/−^ mice were ear-notched using a punch biopsy tool and ear-tagged. DNA was extracted using the KAPA HotStart Mouse Genotyping Kit (Item no. 07961804001, KAPA Biosystems) and CD82^−/−^ genotyping analysis was performed using the KAPA Genotyping kit according the manufacturer’s recommendations. Primers used for genotyping included a common 5′ sequence targeting both the WT and CD82^−/−^ alleles (5′-AGT GGG CCC TGG CTT TCA AC-3′), while allele-specific reverse primer sequences were provided by Dr. C.K. Miranti (antisense WT 5′-GTC ACA GAA CCT GCT GGG AGA G-3′; antisense CD82^−/−^ recognizing the PGKneo targeting cassette 5′-CTA AAG CGC ATG CTC CAG AC-3′). Primers were used at a final concentration of 10 mM and DNA was amplified in a thermocycler under the following conditions: 95 °C for 3 min; 95 °C for 15 s, 60 °C for 15 s, 72 °C for 15 s for 35 cycles, followed by a final extension at 72 °C for 5 min. The WT and CD82^−/−^ primers were run in separate reactions to prevent one band being favored in the amplification. PCR products were then run on a 2% agarose gel and imaged using a Gene Flash biomager (Syngene). The WT band was expected at 300 bp and the CD82^−/−^ band at 180 bp.

*Mdx*^*5cv*^ genotyping was also conducted using the KAPA genotyping kit, with genotyping results confirmed by a Sanger sequencing protocol previously established [26, 27]. Primers used for initial genotyping included forward primer (5′-ATT TGG AAG CTC CCA GAG AC-3′) and reverse primer (5′-TGC TTT AGC TTC AGA AGT CA-3′). DNA was amplified in a thermocycler under the conditions recommended by the manufacturer: 95 °C for 3 min; 95 °C for 15 s, 60 °C for 15 s, 72 °C for 15 s for 35 cycles followed by a final extension step at 72 °C for 5 min. Amplified products were run on a 2% agarose gel and imaged with the Gene Flash bioimager, with the band expected at 250. After confirmation of a visible band, the PCR product was sent to the Boston Children’s Hospital Sequencing Core to obtain the Sanger sequencing which was used to determine the genotype.

### Serum creatine kinase measurements

Serum creatine kinase levels were measured using the Liquid Creatine Kinase Reagent Set from Pointe Scientific [28]. Mice were restrained and 100–150 μL of blood was collected via a survival tail vein nick into BD Microtainer serum separator tubes from age-matched C57BL/6, CD82^−/−^; *mdx*^*5cv*^ and CD82^−/−^:*mdx*^*5cv*^ mice. Blood was allowed to coagulate for 30 min at room temperature and then centrifuged and 5 μL of serum was used in the assay as instructed by the manufacturer. For each blood sample, three measurements were taken and averaged. Values are reported as means and standard errors of the mean.

### In situ evaluation of muscle function

Mice were anesthetized with sodium pentobarbital (80–100 mg/kg body mass) with supplemental doses administered as necessary. The animal was placed on a temperature-controlled (38 °C) test stand (Aurora Scientific model 809B, Aurora, Ontario, Canada) where the knee was stabilized to a horizontal support and the severed tendon of the tibialis anterior (TA) attached to the lever arm of a dual mode muscle lever system (Aurora Scientific model 305C-LR). TA contractions were induced by supramaximal 200 μs square-wave pulses (Aurora Scientific, model 701A muscle stimulator) delivered to platinum electrodes inserted behind the knee near the peroneal nerve. All contractions were initiated at the optimal length (L_o_) for tetanic force and were separated by 60 s intervals to minimize fatigue. Muscles were stimulated with brief trains (150 ms) at frequencies from 20–300 Hz in order to establish the relationship between force and stimulation frequency. During eccentric contractions, the muscle was stimulated for 233 ms at the frequency giving maximum force (200–300 Hz). During the first 100 ms of stimulation, the muscle was held at L_o_ and during the subsequent 133 ms the muscle was lengthened 20% of fiber length at a velocity of 1.5 fiber lengths/s. The muscle was returned to L_o_ 400 ms after the conclusion of the stretch. The eccentric contraction protocol consisted of a series of 5 eccentric contractions that was bracketed by an initial and a final protocol contraction conducted with the muscle maintained at L_o_. The peak force attained during the first 100 ms of the initial, eccentric, and final contractions was used to calculate relative force (force/force on the initial protocol contraction). Specific force was calculated as active tetanic force divided by TA physiological cross-sectional area (pCSA). The pCSA of the TA was calculated as muscle mass divided by the product of FL and muscle density, with fiber length and muscle density taken as 0.60 L_o_ and 1.06 mg/mm^3^, respectively.

### Plethysmography

Plethysmography was performed in the IDDRC Neurobehavioral Core Facility at Boston Children’s Hospital using a whole body SCIREQ TECHNOTE 070 plethysmograph (SCIREQ Respiratory Equipment Inc., Montreal, Canada). The assay studies the ventilatory parameters in conscious, spontaneously breathing subjects by monitoring changes in pressure and flow. Mice were placed in the plethysmograph and allowed to acclimate for 15 min. Measurements, including inspiratory time and tidal volume, were taken on 5 mice/genotype every 15 s for the next 30 min and compared using a *t* test and Prism Graphpad 8.

### Cardiotoxin injury of mice and immunofluorescence staining of Pax7+ cells

Anesthetized C57BL/6 and CD82^−/−^ female mice were injected in the belly of the right tibialis anterior (TA) with 15 μg of cardiotoxin (15 μl volume) per approved institutional protocols, and the left TA was used as a contralateral control, in addition to the quadriceps muscles. Nine mice for each genotype and per each time point were injected with cardiotoxin, collected at 7 or 21 days post injury and frozen in cold isopentane for histological analyses and for determining the number of Pax7+ cells in the tissue. Ten micron frozen sections of tibialis anterior (TA) or quadriceps muscle tissue were taken from the approximate belly of the muscle. For cardiotoxin-injured muscle, sequential sections were taken to locate the injured area. Tissue sections were stained with hematoxylin and eosin as previously described [28]. The size of the injury area was determined by immunofluorescence staining using anti-laminin (Sigma L9393 1:1000) and anti-embryonic myosin heavy chain (clone F1.652 from DSHB, 1:50). The entire tissue sections were imaged and images were stitched together using the Photomerge function in Photoshop (Adobe). Fiber dimensions (minimum Feret’s diameter and myofiber area) were measured using NIH ImageJ.

For detection of satellite cells in tissue sections via Pax7 immunofluorescence analyses, sections were cut at 8 μm and fixed in 4% PFA for 20 min. Sections were washed with TBST (1x TBS with 0.3% Tween 20) 3 x 5 min, followed by blocking with 2.5% normal goat serum for 30 min. Primary antibodies used were Pax7 (Developmental Studies Hybridoma Bank, 334 μg/ml dil 1:20) together with dystrophin (Abcam, ab15277) dil 1:100 or laminin (Sigma L9393) 1:1,000 diluted with TBST and incubated at 4 °C overnight. Slides were washed with TBST 3 x 10 min before adding the appropriate secondary antibodies diluted 1:500 in TBST for 1 h at room temperature. Slides were again washed in TBST 3 x 10 min and mounted in Vectashield/DAPI. Slides were scanned on an Olympus VS120 microscope. Regions of interest were selected from the slide scan and the number of Pax7-positive nuclei and number of fibers (as defined by their outline with dystrophin staining) were manually counted. Per each animal (*n* = 9/group), between 900 and 1300 myofibers were analyzed and the number of Pax7^+^ cells was counted and normalized to the number of regenerating myofibers; comparisons were made using an unpaired Student’s *t* test.

### Primary cell isolations: satellite cells

Satellite cells were isolated using the MACS mouse Satellite Cell Isolation Kit (Item no. 130-104-268, Miltenyi Biotec), followed by positive selection for integrin alpha7-positive cells (Miltenyi item no 130-104-261). Skeletal muscles from C57BL/6 (WT), CD82^−/−^; mdx^5cv^ and CD82^−/−^:*mdx*^*5cv*^ mice were dissected, minced, and dissociated with 5 mg/mL collagenase D and 1.2 U/mL dispase II for approximately 45 min at 37 °C. After complete dissociation, tissue slurry was filtered through a 100-μm filter and centrifuged. Red blood cell lysis was performed using Red Lysis Buffer (Qiagen Item no. 158904, Valencia, CA, USA), filtered through a 40-μm filter and centrifuged again. Myogenic cells were isolated using MACS enrichment columns starting with the Satellite Cell isolation kit (depletion of endothelial and fibroadipogenic progenitors) followed by positive selection for integrin-α7 expressing cells (satellite cells). Satellite cell populations were plated in triplicates on 4 and 8 well Permanox slide chambers coated with recombinant laminin 511(Biogems RL511S) in growth medium (Ham’s F-10 supplemented with 20%FBS, 1× penicillin/streptomycin/glutamine (PSG) and 10 ng/mL bFGF. Slide chambers were fixed in 4%PFA for 20 min at RT at 24, 48, and 72 h and analyzed by immunofluorescence for expression of the myogenic transcription factors Pax7 (DSHB concentrated supernatant, dil 1:200) and MyoD (Santa Cruz m-318, dil 1:100 and MyoD G-1 sc377460), the cell activation marker Ki67 (Cell Signaling 9129S, dil 1;400) and the senescence/pre-apoptosis marker H2A.X (Ser139), also called H2Aγ (Cell Signaling Technology 2577S dil 1:400). Following fixation, cells were briefly rinsed in PBS and permeabilized for 3 min at RT in PBS/0.5% Triton-X. Cells were again washed 2× in 1XPBS and blocked in PBS/10% fetal bovine serum 0.1%Triton X for 45 min (blocking solution). Primary antibodies were diluted in blocking solution and incubated overnight at 4 °C. Slides were washed 3 x 10 min in 1XPBS and incubated with secondary antibodies for 1 h at room temperature. Slides were again washed in 1XPBS as above and mounted using DAPI/Vectashield (VectorLabs). At each timepoint, the percentages of Pax7+MyoD−; Pax7+MyoD+; Pax7+Ki67+; MyoD+ H2Aγ+ cells were counted. A minimum of 100 total cells was counted per each well and timepoint, with 6–10 independent isolations analyzed/genotype. Comparisons of the percentage of cells between genotypes were made using an unpaired Student’s *t* test and GraphPad8 software.

For cell cycle analyses, cells were trypsinized and fixed in ice cold 70% ethanol for 2 h, then stored at − 20 °C until prior to FACS analysis as we previously described [29]. Briefly, cells were spun, washed in 1X PBS, and then incubated for 30 min at RT with a solution of propidium iodide (20 μg/mL) in 1xPBS supplemented with 0.1%Triton X and 0.2 mg/mL DNase-free RNase A. Cells were then washed again in 1XPBS before being analyzed by FACS as previously described [29].

For fusion assays, 50,000 cells/sample were plated in 4-well slide chambers and grown to confluence. Cells were differentiated in DMEM (1 g/L glucose) 2% FBS and 1X ITS (insulin transferrin selenium supplement (ITS-Gibco thermo Scientific 51500-056) for up to 5 days. Cells were fixed in 4%PFA, permeabilized, and blocked as described above. Cells were incubated overnight 4 °C with anti-myosin heavy chain antibody (clone MF-20, DHSB) diluted 1:50. The fusion index was calculated as the percentage of nuclei fused into MHC-positive myotubes/total number of nuclei. Fusion indices were compared between genotypes using a *t* test and GraphPad8 software.

### Isolation of single myofibers

Single myofibers were isolated as previously described [30]. Briefly, extensor digitorum longus muscle was isolated from WT and CD82^−/−^ mice and digested in collagenase D as described [30]. Muscle tissue was mildly digested with Collagenase-DMEM to release single muscle fibers, which were then transferred to 6-well plates and fixed in 4% PFA, pH 7.4, at room temperature (T0). Fibers to be cultured were placed for 48 h in growth medium prior to being fixed in PFA, immunostained and imaged on a Nikon microscope. Comparisons of the percentage of cells between genotypes were made using a *t* test and GraphPad8 software.

### Histological analyses

Muscle tissue was flash frozen in liquid nitrogen-chilled isopentane as described [31]. Sections of 8 μm were taken using a cryostat (Leica Biosystems, Wetzlar, Germany) and subjected to hematoxylin and eosin (H&E). Sections were fixed for 5 min, placed in 70% ethanol followed by rehydration in water. Slides were stained in Harris modified hematoxylin solution (Sigma-Aldrich HHS32) for four and a half minutes, then washed in water before being submerged in eosin Y solution (Sigma-Aldrich HT110116) for a minute and a half, and then washed again in water until clear. The slides were dehydrated in an ascending alcohol series, cleared in xylene, and then mounted with cytoseal (Richard-Allan Scientific™ Cytoseal 60™ Thermo Scientific, 8310-16). All slides were imaged on a brightfield Nikon E800 microscope (Nikon, Tokyo, Japan) using SPOT software (Diagnostic Instruments, Sterling Heights, Michigan, USA). Images were acquired for entire sections and photomerged using Adobe Photoshop. Depending on the depth of the section analyzed, between 505 and 840 myofibers were analyzed per animal (*n* = 6/cohort). Fiber measurements (minimum Feret’s diameter, H&E stains) were analyzed using ImageJ (NIH, Bethesda, Maryland, USA). Statistical analyses were performed using GraphPad Prism 8 (GraphPad Software, San Diego, CA, USA).

### Sirius Red staining

Slides were dehydrated in a descending alcohol series, before being washed in tap water and placed in hematoxylin. Slides were then washed in tap water, Scott’s Blue Water, and then water again before being stained in a 0.1% Sirius Red solution (Rowley Biochemical SO-674). After staining, slides were washed in acidified water and then dehydrated in an ascending alcohol series before being cleared in xylene and mounted with Cytoseal. Images were acquired for entire sections and photomerged using Adobe Photoshop. Merged images were opened in ImageJ and areas with poor staining were cropped out of the image and turned the background color white (<10% of the entire section). The new image was converted to an 8-bit image and thresholded to turn the entire tissue section black. This section was measured as the total tissue area. Images were then thresholded to turn the Sirius Red staining black and the remaining tissue section white. The black area was measured and divided by the total tissue area calculated above to give a percentage of fibrosis in the tissue section. Percentages were compared between genotypes using a *t* test and Graphpad 8.

### Western blotting

Skeletal muscle tissue was snap frozen and ground to a fine powder using a mortar and pestle precooled in liquid nitrogen. The tissue powder was resuspended in RIPA buffer with Protease and Phosphatase Inhibitor Cocktail Set III, EDTA Free (Item nos. 539134-1SET and 524627-1ML, Millipore Sigma). Lysates were sonicated for 3 × 5 s and spun at 13,000 RPM to pellet tissue debris, while the supernatant was collected. Protein concentration was determined using Pierce^TM^ BCA protein assay kit (Thermo Scientific). Samples were made with 4X NUPAGE loading buffer and .05% BME, boiled at 90 °C for 10 min and spun again at 13,000 RPM; the supernatant was loaded onto a 4–12% Nupage Bis-Tris Gel (Item no, NP0322, Invitrogen) or a 4–20% Tris-Glycine gel (Item no. XP04200, Invitrogen), depending on the target protein to be detected. Protein samples were run at 80 V for 10 min followed by 115 V on ice for 1.5 h before being transferred to a nitrocellulose or PVDF membrane using a wet transfer. Membranes were blocked for 1 h at room temperature in 5% non-fat dry milk in TBS-0.1% Tween (TBST) or Odyssey blocking buffer in PBS (LI-COR #927-40000). Primary antibodies (listed in Supplementary Table 1) were diluted as specified and incubated overnight at 4 °C. Membranes were washed three times, 10 min each, with TBST at room temperature, hybridized with an HRP-Conjugated or IRDye 800 (LI-COR #611-132-122) secondary antibody for 1 h at room temperature, and washed with TBST three times for 10 min each. Detection of proteins probed with HRP-conjugated secondary was determined using Western Lightning ® Plus-ECL Enhanced Chemiluminescence Substrate (Item no. NEL103001EA, Perkin Elmer) and blots were exposed on ProSignal^TM^ Blotting Film (Item no. 30-507L, Prometheus Protein Biology Products, Genesee Scientific) and detection of proteins probed with IRDye 800 was done using the Licor Odyssey 9120 Infrared Imaging System. To ensure equal loading of protein, HRP-conjugated blots were stripped using Restore^TM^ Western Blot Stripping Buffer (Item no. 21059, Thermo Scientific) and hybridized with anti-desmin (Item no. ab8592, Abcam); IRDye 800 hybridized blots were normalized with anti-GAPDH polyclonal antibody (Invitrogen #TAB1001). Optical density was measured by FIJI (Image J) Software.

## Results

Our previous study identified CD82 as an effective marker to prospectively isolate human muscle stem cells and found its expression variably decreased in cell lines and muscle tissues from patients with Duchenne muscular dystrophy [14]. To address the functional role of CD82 in normal and dystrophic muscle, we took advantage of a previously generated CD82 knock-out (CD82^*−/−*^) mouse strain, which did not show any gross abnormality [25]. The genotype of CD82^*−/−*^ mice was first confirmed by PCR and RT/PCR (Supplementary Figure 1A) followed by sequence analysis on RNA extracted from WT and CD82^*−/−*^ skeletal muscle. This confirmed the presence of an 83 bp deletion that encompassed exon 2, which includes the ATG start site, as previously reported [25]. Western blot analyses using an antibody previously reported to recognize mouse CD82 (m-35) [9], confirmed the absence of glycosylated and non-glycosylated CD82 proteins (Supplementary Figure 1B). A small 10 kDa band was seen in both WT and CD82^*−/−*^ tissues, suggesting the presence of an alternative product or a small cross-reactive protein. Immunofluorescence studies detected expression of CD82 at the membrane and in cytoplasm of primary mouse myoblasts (Supplementary Figure 1C) and in the cytoplasm of myotubes (Supplementary Figure 1D) of WT animals, including intracellular vesicles. Western blot analyses of differentiating mouse C2C12 lysates detected an increase in expression of CD82 in myotubes up to 10 days following differentiation (Supplementary Figure 1E). Thus, CD82 expression is detected in both mononuclear myogenic cells and in myotubes, although with different subcellular localization.

**Fig. 1:**
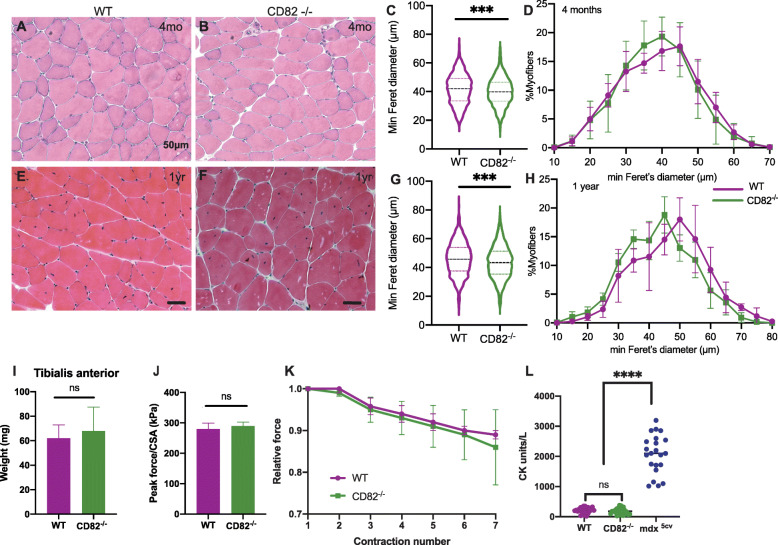
CD82KO skeletal muscle shows no gross histological or functional abnormalities but has smaller myofibers. **a**, **b** H&E representative images of 8-week-old WT and CD82^*−/−*^ *tibialis anterior* (TA) muscles show predominantly normal tissue in both strains. **c** Analyses of myofiber size via minimum Feret’s diameter measurements show significant difference in myofiber size between the two strains. Between 505 and 840 myofibers were measured per each animal, *n* = 6 animals/genotype. **d** Myofiber distribution plots of WT and CD82^*−/−*^ at 8 weeks. **e**, **f** H&E representative images of 1-year-old WT and CD82^*−/−*^ TA muscles, respectively. **g** Analyses of myofiber size via minimum Feret’s diameter measurements show significant difference between the two strains. **h** Myofiber distribution plots of WT and CD82^*−/−*^ at 1 year. **i** Comparison of TA weight does not show significant differences between genotypes. **j** Peak force measurements normalized to CSA and **k** relative force measurements following serial tetanic stimulations do not show significant differences between genotypes. **l** Serum creatine kinase levels are normal and undistinguishable between WT and CD82^−/−^ mice. Sera from *mdx*^*5cv*^ mice were used as positive controls. Each dot represents an independent animal. ***p < 0.001; ****p < 0.0001. *N* = 6–10 animals/genotype in each experiment, with the exception of CK analyses (**l**), where a minimum of 20 mice were analyzed per genotype

**Fig. 2:**
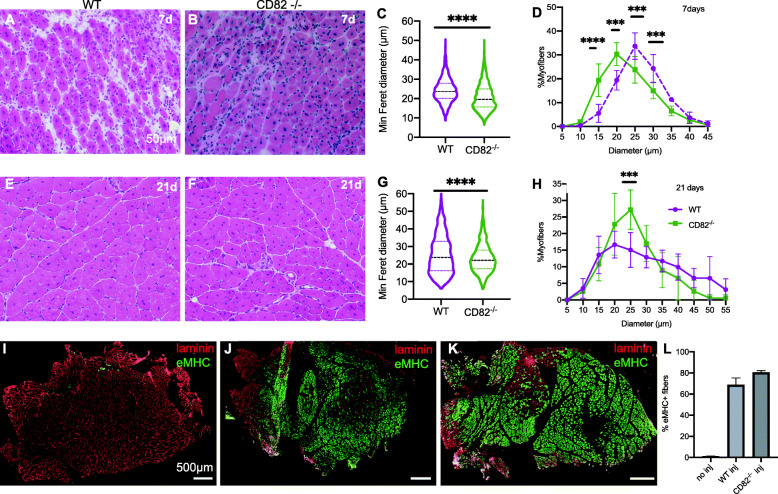
CD82KO skeletal muscle shows delayed regeneration with smaller myofibers. **a**, **b** H&E representative images of regenerating WT and CD82^*−/−*^ muscles at 7 days. Comparisons of myofiber size at 7 days following CTX injury via minimum Feret’s diameter measurements (**c**) and myofiber distribution plots (**d**) show significant differences between the two strains using *t* tests. **e**, **f** H&E representative images of regenerating WT and CD82^*−/−*^ muscles at 21 days. Minimum Feret’s diameter comparisons (**g**) and myofiber distribution plots (**h**) at 21 days following CTX injury show significant differences between the two strains using *t* tests (**i**–**k**) Immunofluorescence images of entire sections stained for laminin (red) and embryonic MHC, which labels regenerating fibers (green). **i** is a control immunostaining of non-injured CD82^*−/−*^ muscle (no e-MHC+ fibers), whereas **j** and **k** are WT and CD82^*−/−*^ injured muscles 7 days following injury, respectively. **l** Graph of percentages of regenerating fibers in non-injured and cardiotoxin injured muscles. ***p < 0.001; ****p < 0.0001

To study the function of CD82 under basal (non-dystrophic) conditions, in situ evaluation of muscle force, serum creatine kinase levels, muscle mass, and histological assessment analyses were performed on CD82^−/−^ mice compared to age-matched WT controls. H&E stain under basal conditions did not show any gross histological abnormality between WT and CD82^−/−^ skeletal muscle (Fig. 1a, b). Average myofiber sizes measured via minimum Feret’s diameter and compared via *t* test showed a significant decrease in CD82^−/−^ compared to WT myofibers both at 2-month-old (Fig. 1c) and 1-year-old mouse cohorts (Fig. 1g). Frequency distribution plots at 2 months and 1 year of age did not show significant differences between the genotypes (Fig. 1d, h). The weight of the TA muscle did not appear to significantly differ between WT and CD82^−/−^ animals (Fig. 1i), nor assessments of peak force normalized to the cross sectional area of muscles (Fig. 1j), force generated following serial eccentric contractions (Fig. 1k), or serum CK values (Fig. 1l), which appeared normal in CD82^−/−^ mice compared to WT controls, while *mdx*^*5*cv^ sera were used as a positive control for the CK assay (Fig. 1l). Histological analyses were also performed under regenerating conditions (Fig. 2), where 9 WT and 9 CD82^−/−^ TA muscles were injured with cardiotoxin and allowed to undergo repair for 7 or 21 days. Following injury, CD82^−/−^ muscle presented with significantly delayed regeneration and reduced myofiber size at both 7 days following injury (Fig. 2a, b, quantifications in c, d) and at 21 days (Fig. 2e, f, quantifications in g, h). The overall size of muscle injury was assessed by staining for laminin and embryonic myosin in non-injured CD82^−/−^ muscle (negative control, Fig. 2i), injured WT (Fig. 2j), and injured CD82^−/−^ (Fig. 2k) muscles (quantification in 2L). To address whether the delayed regenerative capacity of CD82^*−/−*^ muscle was due to a decreased number of satellite cells, the number of Pax7-expressing cells was analyzed in control and CD82^*−/−*^ muscle tissue sections using both uninjured and regenerating muscle. Per each animal (*n* = 9/group), between 900 and 1300 myofibers stained for dystrophin were analyzed for presence of adjacent Pax7^+^ satellite cells. No significant difference was found in the total number of Pax7^+^ cells under basal or regenerating conditions (Supplementary Figure 2). To further confirm this data, myofiber explants of EDL muscles were obtained from WT and CD82^*−/−*^ mice and single myofibers were immunostained for Pax7, which confirmed the number of Pax7^+^ cells did not differ between the genotypes (Supplementary Figure 2, bottom panels). These results concluded that the impairment in muscle regeneration observed in injured CD82^*−/−*^ muscles is not due to a decrease in Pax7^+^ satellite cell number.

**Fig. 3:**
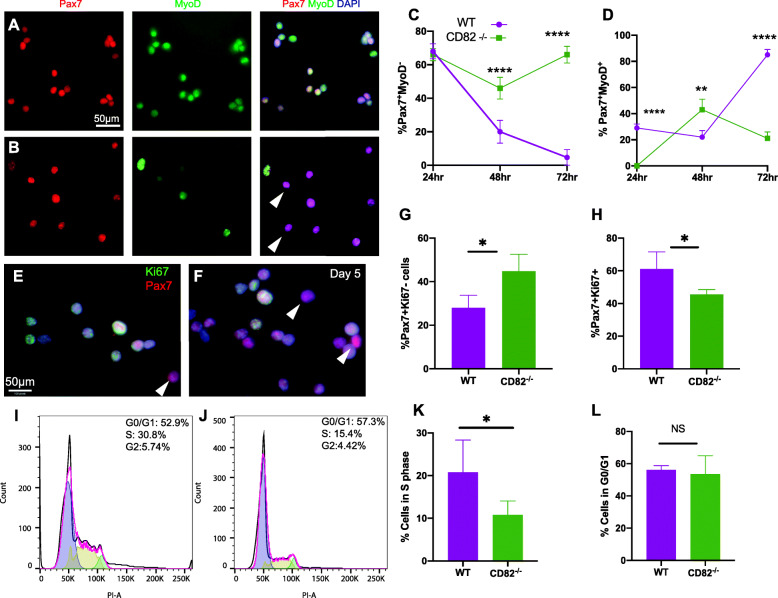
Satellite cell activation is transient in CD82^*−/−*^ mice and Pax7 expression is retained. **a**, **b** Immunofluorescence staining of satellite cells for Pax7 (red) and MyoD (green) 72 h post-extraction from WT (**a**) and CD82^*−/−*^ (**b**) animals, respectively (*n* = 6–10/genotype). Arrowhead in (**b**) points to Pax7^+^MyoD^-^ cells that are more abundant in CD82^*−/−*^ cultures. **c** Quantification of quiescent Pax7^+^MyoD^−^ satellite cells at 24, 48, and 72 h following isolation and **d** Pax7^+^MyoD^+^ activated satellite cells from WT and CD82^*−/−*^ cultures at the same timepoints (**p ≤ 0.01; ****p ≤ 0.0001). **e**–**h** Images and quantification of Pax7^+^Ki67^*−*^ (**g**) and Pax7^+^Ki67^+^ (**h**) cells extracted from WT (**e**) and CD82^*−/−*^ (**f**) cultures 5 days following isolation (*p < 0.05). Arrowheads in **e** and **f** point at Pax7^+^Ki67^*−*^ cells, which are more abundant in CD82^*−/−*^ cultures. **i**, **j** Flow cytometry cell cycle profiles of WT and CD82^*−/−*^ myogenic cultures following expansion for ~ 30 days. 10,000 events were analyzed per each sample via FACS. **k** Quantification of percentage of cells in the S-phase and **l** in the G0/G1 phase from WT and CD82^*−/−*^ cultures (*p ≤ 0.05; NS = not significant)

To determine if CD82^−*/*−^ satellite cells exhibited impairment in activation and/or cell division, primary myogenic cultures were established (Fig. 3). Satellite cells were extracted from pooled skeletal muscles of WT or CD82^*−/−*^ animals using an established two-step magnetic column enrichment protocol, first via depletion of fibro-adipogenic and endothelial progenitors followed by positive selection of α7-integrin expressing cells (Miltenyi Biotec) [32]. Cells were plated in slide chambers and cultured in growth medium supplemented with bFGF to promote activation (*n* = 6-10 isolations/genotype). At 24, 48, and 72 h following isolation, cells were fixed and immunostained for Pax7 with MyoD and Pax7 with Ki67 to determine the percentage of quiescent and activated satellite cells, while co-staining of MyoD with H2Aγ was used to determine the percentage of myogenic cells undergoing premature senescence and in a pre-apoptotic state, defined by increased phosphorylation at Ser 139 of H2A.X [33] (Supplementary Figure 3A-D). Co-expression of MyoD and H2Aγ showed no significant difference in senescent/pre-apoptotic cell number between WT and CD82^*−/−*^ cultures at all timepoints analyzed (Supplementary Figure 3A-C). Consistent with these findings, the number of non-adherent cells in the cultures did not differ between the two genotypes at each timepoint (Supplementary Figure 3D). Percentages of Pax7^+^MyoD^-^ and Pax7^+^MyoD^+^ cells were calculated at 24, 48, and 72 h post isolation and compared between genotypes. Compared to WT cultures (Fig. 3a), CD82^*−/−*^ cultures (Fig. 3b) contained significantly more Pax7^+^MyoD^-^ cells at both 48 and 72 h following isolation (Fig. 3c) and significantly less Pax7^+^MyoD^+^ cells at 0 and 72 h (Fig. 3d). Notably, at 48 h, a significant increase in Pax^+^MyoD^+^ activated satellite cells was observed in CD82^−*/*−^ cultures compared to WT, which was completely reversed at 72 h, when ~ 20% Pax^+^MyoD^+^ cells were observed in CD82^*−/−*^ cultures, compared to ~ 80% in WT ones (Fig. 3d). Conversely, ~ 5% of quiescent Pax7+MyoD− were seen at 72 h in WT cultures, while the percentage of quiescent (inactive) Pax7+MyoD− cells sharply increased in CD82^*−/−*^ cultures to > 60% (Fig. 3c). These findings indicated that loss of CD82 expression results in transient activation of the satellite cells that is not sustained*.* In addition, the percentage of Pax7^+^Ki67^−^ and Pax7^+^Ki67^+^ cells were also examined and compared between genotypes at day 5 from isolation. Interestingly, CD82^−*/*−^ cultures contained significantly more Pax7^+^Ki67^-^ cells (Fig. 3f, g) and significantly less Pax7^+^Ki67^+^ cells (Fig. 3h) when compared to WT cultures (Fig. 3e), confirming delayed and/or decreased satellite cell activation. Cell cycle analyses indicated that CD82^−*/*−^ cultures had less cells in the S-phase compared to WT cultures (Fig. 3i–k); however, the percentage of cells in the G0/G1 phases did not differ between the two genotypes (Fig. 3l). Furthermore, when myogenic cultures were induced to differentiate, the fusion index was significantly lower in CD82^*−/−*^ cultures compared to WT controls at day 5 (*p* < 0.0001; Supplementary Figure 3E-N). Collectively, these studies suggest that satellite cell activation and differentiation/fusion are impaired or delayed in CD82^−*/*−^ compared to WT muscles.

**Fig. 4:**
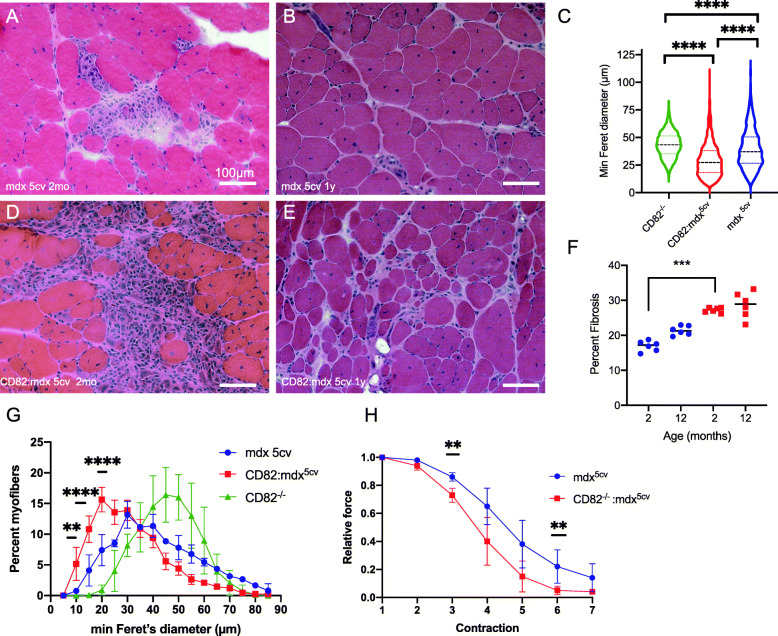
Ablation of CD82 in dystrophic muscle leads to worsened skeletal muscle phenotype. H&E images of the quadriceps muscles from *mdx*^*5cv*^ (**a**, **b**) and from CD82^*−*/*−*^:*mdx*^*5cv*^ mice (**d**, **e**). Images were taken from tissues at 2 months (**a**, **d**) and 1 year (**b**, **e**) of age. **c** Comparisons of myofiber size using ANOVA shows significantly smaller myofibers in both strains of dystrophic mice compared to CD82^−/−^ mice, with CD82^−/−^:*mdx*^*5cv*^ myofibers being the smallest. **f** Quantification of fibrosis at 2 months and 1 year of age shows increased presence of fibrotic tissue in young CD82^*−*/*−*^:*mdx*^*5cv*^ compared to control *mdx*^*5cv*^ mice. Examples of images used in quantification are shown in Supplementary Figure 4F, G. **g** Distribution plots of myofiber size show significantly smaller myofibers in CD82^*−*/*−*^:*mdx*^*5cv*^ compared to control *mdx*^*5cv*^ and CD82^*−*/*−*^ muscles. **h** Isometric force analysis following serial eccentric contractions demonstrated significantly decreased muscle force production after each contraction in CD82^*−*/*−*^:*mdx*^*5cv*^ compared to control *mdx*^*5cv*^ mice. *N* = 6/group (**p ≤ 0.01; ***p < 0.001; ****p < 0.0001). Scale bars: 100 μm

To establish the effect of loss of CD82 expression in dystrophic muscle, CD82^−/−^:*mdx*^*5cv*^ mice were generated and aged up to 1 year, with comparisons made between *mdx*^*5cv*^ and CD82^−/−^:*mdx*^*5cv*^ cohorts. CD82^−/−^:*mdx*^*5cv*^ exhibited severe kyphosis compared to *mdx*^*5cv*^ controls (Supplementary Figure 4A, B). Pulmonary function tests (plethysmography) demonstrated that CD82^−/−^:*mdx*^*5cv*^ have significantly increased inspiration time compared to *mdx*^*5cv*^ mice, confirming impaired pulmonary function (Supplementary Figure 4C) [34]. Histological H&E analyses performed on whole sections of quadriceps at 2 months (Fig. 4a, d, quantification in Supplementary Figure 4D) showed significantly decreased myofiber size in CD82^−/−^:*mdx*^*5cv*^ compared to control *mdx*^*5cv*^ and the significance increased at 1 year of age (Fig. 4b, e; quantifications in c, g), including comparisons with CD82^−/−^ muscle (non-dystrophic). Quantification of fibrotic areas was also performed at 2 months and 1 year of age in both genotypes (Fig. 4f), which showed significantly increased fibrosis in CD82^−/−^:*mdx*^*5cv*^ mice, accompanied by increased serum CK levels at this timepoint (Supplementary Figure 4E). Isometric force analyses following serial eccentric contractions demonstrated significantly heightened susceptibility to force loss, an indication of greater acute muscle injury following each eccentric contraction in CD82^−/−^:*mdx*^*5cv*^ compared to control *mdx*^*5cv*^ mice (Fig. 4h).

**Fig. 5:**
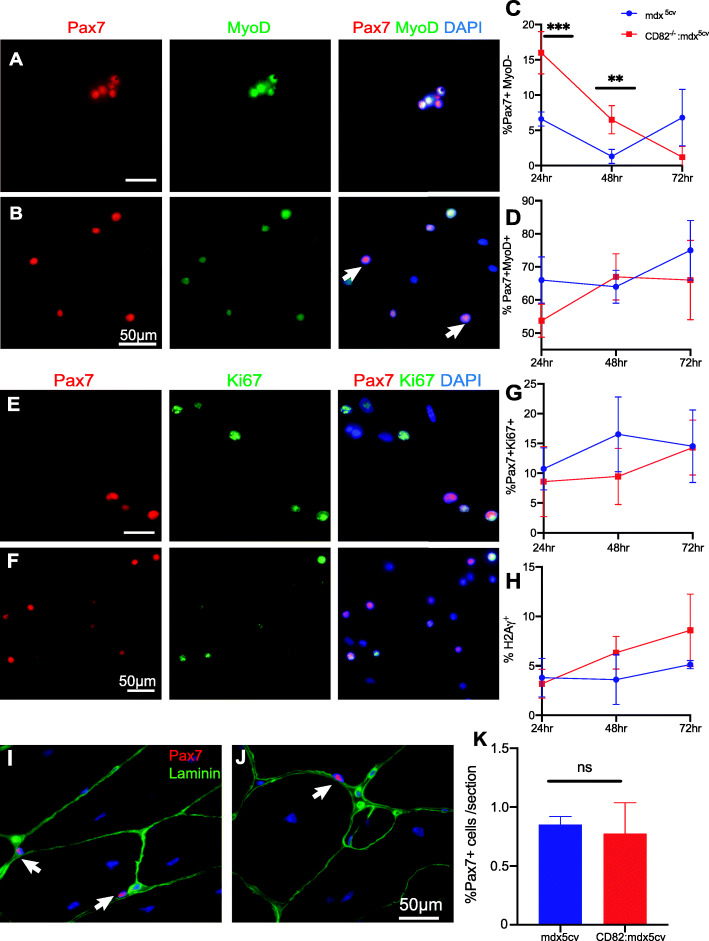
CD82^*−*/*−*^:*mdx*^*5cv*^ satellite cells exhibit decreased activation potential compared to *mdx*^*5cv*^. Satellite cell-derived cultures from *mdx*^*5cv*^ (**a**) and CD82^*−*/*−*^:*mdx*^*5cv*^ (**b**) muscles stained for Pax7 (red) and MyoD (green) 72 h post isolation. Quantification plots of Pax7^+^MyoD^*−*^ (**c**) and Pax7^+^MyoD^+^ (**d**) cells at 24, 48, and 72 h post isolation in *mdx*^*5cv*^ and CD82^*−*/*−*^:*mdx*^*5cv*^ revealed significant increase in Pax7^+^MyoD^*−*^ cells at 72 h post isolation in CD82^*−*/*−*^:*mdx*^*5cv*^ cultures (***p < 0.001). Immunofluorescence staining of primary cultures from *mdx*^*5cv*^ (**e**) and CD82^*−*/*−*^:*mdx*^*5cv*^ (**f**) mice using Pax7 (red) and Ki67 (green). Nuclei were counterstained with DAPI. **g** Quantification of Pax7^+^Ki67^+^ and **h** H2Aγ cells in *mdx*^*5cv*^ and CD82^*−*/*−*^:*mdx*^*5cv*^ cultures revealed no significant differences at 24, 48, or 72 h post extraction. **i**–**k** Immunofluorescence staining and quantification of Pax7^+^ satellite cells in tissue sections from *mdx*^*5cv*^ and CD82^*−*/*−*^:*mdx*^*5cv*^ mice. Pax7 staining is in red and laminin staining is in green. Satellite cells were counted in entire sections of the quadriceps but no significant difference between genotypes was observed. *N* = 6–10/genotype. Scale bars: 50 μm

To determine whether a chronic demand for satellite cell activation in dystrophic muscle could explain the worsened phenotype of CD82^−/−^:*mdx*^*5cv*^, satellite cells from CD82^−/−^:*mdx*^*5cv*^ and *mdx*^*5cv*^ mice were extracted and analyzed in parallel for presence of quiescent and activated cells. Cells were again plated in slide chambers and cultured in growth medium as described for WT and CD82^−/−^ cultures. At 24, 48, and 72 h following isolation, cells were fixed and immunostained for Pax7 with MyoD and Pax7 with Ki67 to determine the percentage of quiescent and activated satellite cells, while co-staining of MyoD with H2Aγ was used to determine the percentage of myogenic cells undergoing premature senescence/apoptosis (Fig. 5). The percentage of Pax7^+^MyoD^-^ cells was significantly increased in CD82^−/−^:*mdx*^*5cv*^ compared to *mdx*^*5cv*^ cultures at 72 h (Fig. 5a, b, quantification in c), while the percentages of Pax7^+^MyoD^+^ (Fig. 5d), Pax7^+^Ki67^+^ (Fig. 5g), and MyoD^+^H2Aγ^+^ (Fig. 5h) did not differ between the genotypes at any timepoint analyzed, nor the number of non-adherent cells at each timepoint (Supplementary Figure 5D), indicating that adhesion and apoptosis were likely not increased in cultures lacking CD82. The percentage of Pax7^+^ satellite cells was also analyzed in tissue sections by immunofluorescence (Fig. 5i, j) in *mdx*^*5cv*^ and CD82^−/−^:*mdx*^*5cv*^ co-stained with laminin, to highlight the myofiber basal lamina. The percentage of Pax7^+^ cells in tissue sections did not differ between the genotypes (Fig. 5k). Myoblasts from *mdx*^*5cv*^ and CD82^−/−^:*mdx*^*5cv*^ mice were also induced to differentiate (Supplementary Figure 5A, B) and the fusion index at day 5 was significantly lower in CD82^−/−^:*mdx*^*5cv*^ cultures (*p* < 0.05; Supplementary Figure 5C). Thus, loss of CD82 expression appears to affect/delay activation of satellite cells, with more cells likely to return to an inactive state, defined as Pax7^+^MyoD^−^ by protein expression.

**Fig. 6:**
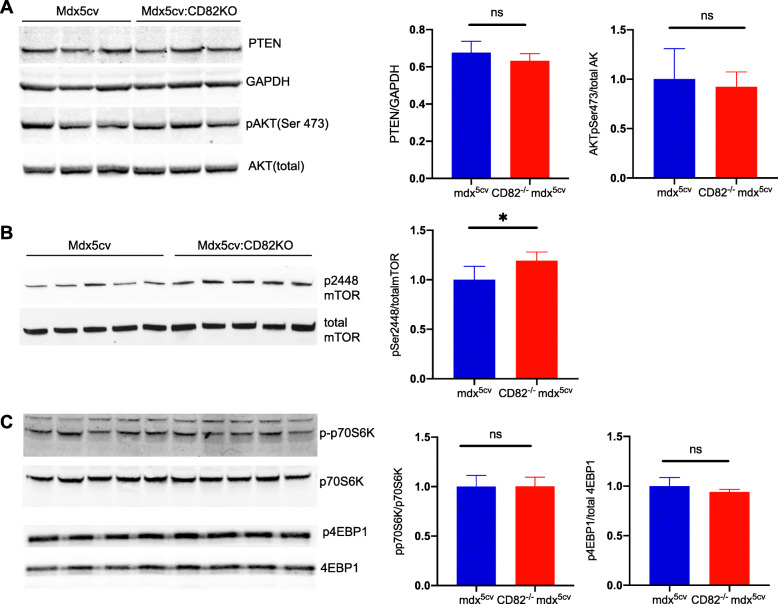
Loss of CD82 expression in dystrophic muscle leads to increased mTOR phosphorylation at Ser 2448. Quadriceps muscle extracts from 12-week-old male *mdx*^*5cv*^ and CD82^*−*/*−*^:*mdx*^*5cv*^ mice were analyzed by Western blot and probed for multiple signaling proteins known to control cell size and quantified. **a** Quantification of PTEN and pAKT (Ser 473) protein levels did not show significant differences between *mdx*^*5cv*^ and CD82^*−*/*−*^:*mdx*^*5cv*^ muscle extracts. **b** Immunoblots for mTOR (pSer 2448) and mTOR (total) show an increase of mTOR-phosphoSer2448 in CD82^*−*/*−*^:*mdx*^*5cv*^ compared to *mdx*^*5cv*^ muscles extracts (*p = 0.02). **c** Analyses of p-p70S6K and p4EBP1, two known targets of mTOR signaling normalized to their total non-phosphorylated forms show no significant differences between the genotypes. N = 5/genotype

To study the downstream mechanism(s) responsible for decreased myofiber size and strength in dystrophic animals where CD82 expression was ablated, we examined the expression of proteins upstream and downstream of the mTOR/AKT signaling pathway, a major regulator of cell growth, protein synthesis, cellular proliferation, and cell survival in many tissues, including skeletal muscle [35]. PTEN is a known negative regulator of AKT phosphorylation and its expression levels were similar in CD82^−/−^:*mdx*^*5cv*^ compared to *mdx*^*5cv*^ muscle (Fig. 6a). The phosphorylation levels of AKT at Ser473 were also assayed by western blot, which also showed that phosphorylation at Ser473 did not differ in CD82^−/−^:*mdx*^*5cv*^ muscle compared to control *mdx*^*5cv*^ (Fig. 6a). mTOR is an important regulator of protein synthesis downstream of AKT and two known mTOR targets are 4EBP1 and S6K. We found an increase in mTOR phosphorylation at Ser 2448 in dystrophic mice lacking CD82 (Fig. 6b), while 4EBP1 and p70S6K and their respective phosphorylated forms did not differ between the genotypes (Fig. 6c).

## Discussion

Tetraspanins are a heterogeneous and ubiquitous group of membrane-bound proteins that regulate a variety of physiological processes, from stem cell function to tissue regeneration and cancer progression [2, 4, 5]. Tetraspanins are known to associate with specific non-tetraspanin proteins and by doing so, they regulate membrane compartmentalization in a highly hierarchical and cell type-specific manner [3]. However, how this network of hierarchical protein interactions is established and maintained is not well understood. Several tetraspanins are expressed in skeletal muscle, such as CD81 and CD53, which have been reported to play a role in differentiation and fusion of muscle cells [36, 37]. More recently, the tetraspanin KAI/CD82 was shown to be a prospective marker for the isolation of human muscle stem cells [14] and its expression is retained by myoblasts upon differentiation into myotubes. In myogenic cells, CD82 specifically interacts with α7 integrin and the dystrophin-associated protein α-sarcoglycan [14]. Importantly, CD82 expression is reduced in cell lines and muscle tissue from Duchenne muscular dystrophy (DMD) patients, suggesting that its expression is affected by loss of dystrophin [14]. However, the function of CD82 in dystrophic muscle and whether its decreased expression is a consequence of loss of dystrophin or a compensatory mechanism in response to loss of dystrophin remains to be elucidated.

In the present study, we determined the consequences of CD82 loss-of-function in both normal and dystrophic skeletal muscle. Our data support the conclusion that CD82 is necessary for proper activation of satellite cells, as shown by both in vitro and in vivo data comparing WT and CD82^−/−^ genotypes. Moreover, when CD82 is depleted in a dystrophic background, where muscle regeneration is continuously occurring and in demand, the disease is significantly worsened. In the absence of muscle insult, there is no significant histological change between WT and CD82^−/−^ skeletal muscle. The importance of CD82 in muscle regeneration is demonstrated by the delayed regenerative capacity of CD82^−/−^ muscles during acute cardiotoxin injury, as well as during chronic injury due to muscle disease, when CD82 expression was removed in the *mdx*^*5cv*^ mouse.

Muscle repair is largely dependent on the proper function of muscle stem cells, the satellite cells [38–40]. In muscles lacking expression of CD82, regardless of whether our studies were performed in normal or dystrophic background, satellite cells appeared normal in number, but reluctant to divide, while retaining a seemingly quiescent, inactive state (Pax7^+^MyoD^−^). This reluctance to activate resulted in poor progression of muscle cells into a mature state, accompanied by reduced in vitro fusion capacity, which presumably lead to decreased myofiber size, poor regenerative capacity in vivo, and physiologically weakened myofibers with heightened susceptibility to injury when compared to *mdx*^*5cv*^ controls. The severe decrease in satellite cell activation following loss of CD82 expression differs from what is reported for bone marrow stem cells, where loss of CD82 expression leads to hyperactivation and increased cell division [20, 21]. Thus, CD82 function is likely regulated through different binding partners and/or through different downstream effectors in muscle cells compared to hematopoietic stem cells (HSCs), as expected for tetraspanins.

Decreased myofiber size can arise through multiple mechanisms, including decreased myogenic cell fusion and/or decreased muscle protein synthesis. Our studies highlight possible contributions from both mechanisms following loss of CD82. Myogenic cells from CD82^−/−^ muscle exhibit significantly decreased cell fusion in vitro, suggesting that CD82 might be directly or indirectly linked to proteins responsible for promoting fusion. Membrane-membrane fusion is one well-described function of tetraspanins, which include CD9 as a critical regulator of sperm-egg fusion [41–44]. In skeletal muscle CD9 and CD81 have been shown to negatively regulate myoblast fusion [45]. Thus, different tetraspanins might be regulating myoblast fusion and myofiber growth through antagonistic functions: CD82 and CD53 [36] being positive regulators of myoblast fusion, while CD9 and CD81 are inhibitors [45]. Collectively, these studies point to the importance of determining if expression of CD9 and CD81 is temporally preceding the expression of CD82 and CD53 during myogenic differentiation and fusion of myogenic cells, which would indicate that CD9 and CD81 are necessary to prevent myogenic cells from premature fusion. Previous studies including our own have shown that CD82 is expressed in both satellite cells and downstream myoblasts and myotubes, which leads to the fascinating hypothesis that multiple tetraspanins might be co-expressed at the same time and that regulation of myogenic fusion might occur through binding and assembly of diverse protein complexes with antagonistic function, potentially including the recently identified bona fide proteins necessary for myogenic fusion myomaker and myomerger [46].

Distinct from myoblast fusion, the present study found that dysregulation of the PTEN/AKT pathway downstream of CD82 is not responsible for the reduced myofiber size observed in CD82^−/−^ and CD82^−/−^:*mdx*^*5cv*^ dystrophic muscles. Deletion of PTEN in satellite cells has been shown to impair the ability of satellite cells to self-renew [47], which was accompanied by an increase in AKT phosphorylation at Ser473 and an increase in protein synthesis through phosphorylation of ribosomal S6 kinase, a target of mTOR/AKT [47]. Downstream of AKT, mTOR is an important regulator of protein synthesis and regulation of autophagy and decreased mTOR signaling in skeletal muscle leads to severe myopathy [48]. Interestingly, our study demonstrates an increase in mTOR phosphorylation at Ser 2448 in dystrophic mice lacking CD82, while no significant differences were seen in other known downstream targets. Increased phosphorylation of mTOR Ser2448 has been observed specifically in the skeletal muscle of lamin-A deficient mice, which are characterized by skeletal muscle dystrophy and dilated cardiomyopathy [49]. While previous studies have implicated this site as a measure of mTOR activity, recent findings also point to a possible negative feedback loop mechanism as a consequence of p70S6K activity with the ultimate effect of inhibiting rather than activating mTOR [50], indicating the complex and diverse regulation involving mTOR phosphorylation at this particular site.

## Conclusions

In summary, we have identified that CD82 plays a positive function in skeletal muscle regeneration. Loss of CD82 expression in skeletal muscle causes decreased/delayed activation of satellite cells, accompanied by decreased proliferation. Loss of CD82 negatively impacts muscle regeneration, leading to a more severe manifestation of the dystrophic symptoms in CD82^−/−^:*mdx*^*5cv*^ mice. Collectively, these findings suggest that upregulation of CD82 may be of therapeutic benefit in diseased muscle, including dystrophic muscle. Future studies will address the safety and potential benefits of CD82 upregulation in satellite cells or in myofibers and determine whether similar or distinct effects might be observed when CD82 upregulation is driven in different subcellular entities of skeletal muscle.

## Supplementary Information

**Additional file 1:** **Supplementary Figure 1.** Confirmation of CD82 knockout mice at RNA and protein levels and detection of CD82 expression in murine myoblasts and myotubes. A) Ethidium bromide gel staining showing amplification of CD82 cDNA from WT and CD82^-/-^ mice. B) western blot analyses of 2WT and 2CD82^-/-^ skeletal muscle tissue lysates showing expression of glycosylated and non- glycosylated CD82 protein in WT tissue, which is absent in CD82^-/-^ extracts. A small band at ~10 Kda was seen in both WT and CD82^*-/-*^ tissues, suggesting presence of an alternative CD82 product or a small cross-reactive protein. GAPDH detection was used as total protein loading control. (C, D) Immunofluorescence staining of CD82 (green) and MyoD (red) in WT mouse primary myoblasts (C) and myotubes (D). CD82 expression is detected in both myoblasts and myotubes, with increased expression in the cytoplasm of myotubes in vesicles. E) Western blot of a time-course differentiation of C2C12 myogenic cells up to 10 days, showing expression of CD82 increases during differentiation. GAPDH detection is used as total protein loading control.

**Additional file 2:** **Supplementary Figure 2.** The number of Pax7+ satellite cells does not differ between WT and CD82^-/-^ animals. Upper panels show single stained and merged representative immunofluorescence images of TA muscle tissue sections from non-injured (WT and CD82^*-/-*^) and cardiotoxin-injured muscles 7 days following injury (WT CTX7D and CD82^*-/-*^CTX7D) mice. Sections were immunostained for Pax7 (green) dystrophin (red) and DAPI (blue) as indicated. Arrows point to nuclei of Pax7+ cells in each panel. Quantification of the number of Pax7+ cells/myofiber from uninjured muscle and from regenerating muscle show no significant difference between the genotypes. Scale bars=50 microns. Lower panels show single myofibers isolated from EDL muscles of WT and CD82^*-/-*^ mice immediately fixed and stained for Pax7 expression (red). Quantification of the number of Pax7+ cells per single myofibers between WT and CD82^*-/-*^show no significant difference (n=4/genotype).

**Additional file 3: Supplementary Figure 3.** Delayed fusion in CD82^-/-^ myoblast cultures is not due to early apoptosis/or reduced cell adhesion. (A, B) Immunofluorescence staining of cultures for MyoD (red) and H2Aγ (green) 72 hrs post-extraction from WT (A) and CD82^*-/-*^ (B) animals. Arrowhead in (A) points at a MyoD^+^H2Aγ^+^ cell. (C) Quantification of H2Aγ^+^ cells and (D) non-adherent cells revealed no significant differences between the genotypes at all timepoints analyzed. (E) Myogenic cells from WT and (F) CD82^*-/-*^ mice were plated at the same density and induced to form myotubes. At day 3 and 5 following differentiation, WT and CD82^*-/-*^ cultures were immunostained for myosin heavy chain (red staining in G, H, L, M) to quantify the fusion index. The purity of the cultures assessed by MyoD staining was >80-90%. At day 3 there was no difference in myotube formation between cultures, as assessed by fusion index following staining with MF20 (I). (N) Quantification of fusion index after 5 days in differentiation media showed significantly decreased fusion in CD82^*-/-*^ compared to WT cultures. Fusion index was calculated as the ratio of number of nuclei fused in MHC-myotubes over the number of total nuclei (****p<0.0001).

**Additional file 4:** **Supplementary Figure 4.** CD82^-/-^:*mdx*^*5cv*^ mice are more severely affected than *mdx*^*5cv*^ at early age. (A) Representative image of *mdx*^*5cv*^ and (B) CD82^-/-^:*mdx*^*5cv*^ mice at 1 year of age. CD82^-/-^:*mdx*^*5cv*^ mice show severe kyphosis. (C) Plethysmography assay shows significantly increased inspiration time in CD82^-/-^:*mdx*^*5cv*^ compared to *mdx*^*5cv*^ controls (***p<0.001). (D) Distribution plot of percentage of myofibers in CD82^-/-^:*mdx*^*5cv*^ (red) and *mdx*^*5cv*^ (blue) with minimum Feret diameter ranging from 10 to 90 microns. The plot shows how CD82^-/-^:*mdx*^*5cv*^ mice have significantly smaller myofibers at 2 months of age. Comparisons were made with multiple t-tests (*=p≤0.05; **p≤0.01). (E) Serum creatine kinase (CK) activity assays were performed over a period of 20 weeks in independent cohorts of mice (n=4-8/timepoint/genotype). Multiple t-test analysis was made to compare the genotypes at each timepoint. The most significant changes in membrane permeability in CD82^-/-^:*mdx*^*5cv*^ compared to control *mdx*^*5cv*^ mice were observed between 5 and 10 weeks of age. (F-G) Examples of merged images of Sirius red stains used to quantify fibrotic tissue in *mdx*^*5cv*^ and CD82^-/-^:*mdx*^*5cv*^ mice at 2 months and one year. The images shown are examples of muscles at 2 months of age. Quantifications are shown in Fig. 4f.

**Additional file 5:** **Supplementary Figure 5.** (A, B) Myogenic cells from *mdx*^*5cv*^ and CD82^-/-^:*mdx*^*5cv*^ mice were plated at the same density and induced to form myotubes. At day 5 following differentiation, cultures were immunostained for myosin heavy chain (red) to quantify the fusion index. (C) Quantification of fusion index shows significantly decreased fusion in CD82^-/-^:*mdx*^*5cv*^ compared to *mdx*^*5cv*^ cultures *p<0.05. (D) Quantification of non-adherent cells in *mdx*^*5cv*^ and CD82^-/-^:*mdx*^*5cv*^ cultures show no significant difference in their number up to 72 hrs following isolation.

**Additional file 6:** **Supplementary Table 1.** List of primary antibodies used in this study.

## Data Availability

All data presented in this article are available from the corresponding author upon reasonable request.

## Abbreviations

DMD: Duchenne muscular dystrophy
CK: Creatine kinase
CTX: Cardiotoxin
mTOR: Mammalian target of rapamycin
pSer: Phospho-Serine
AKT: Protein kinase B
