# Supplementary material for: Tetraspanin CD82 is necessary for muscle stem cell activation and supports dystrophic muscle function
Source: Skelet Muscle. 2020 Nov 27;10:34. doi: 10.1186/s13395-020-00252-3 (PMC7693590; doi:10.1186/s13395-020-00252-3)
Supplement: Supplementary file 1 — Additional file 1: Supplementary Figure 1. Confirmation of CD82 knockout mice at RNA and protein levels and detection of CD82 expression in murine myoblasts and myotubes. A) Ethidium bromide gel staining showing amplification of CD82 cDNA from WT and CD82-/- mice. B) western blot analyses of 2WT and 2CD82-/- skeletal muscle tissue lysates showing expression of glycosylated and non- glycosylated CD82 protein in WT tissue, which is absent in CD82-/- extracts. A small band at ~10 Kda was seen in both WT and CD82-/- tissues, suggesting presence of an alternative CD82 product or a small cross-reactive protein. GAPDH detection was used as total protein loading control. (C, D) Immunofluorescence staining of CD82 (green) and MyoD (red) in WT mouse primary myoblasts (C) and myotubes (D). CD82 expression is detected in both myoblasts and myotubes, with increased expression in the cytoplasm of myotubes in vesicles. E) Western blot of a time-course differentiation of C2C12 myogenic cells up to 10 days, showing expression of CD82 increases during differentiation. GAPDH detection is used as total protein loading control. [file 13395_2020_252_MOESM1_ESM.pptx]

## Slide 1
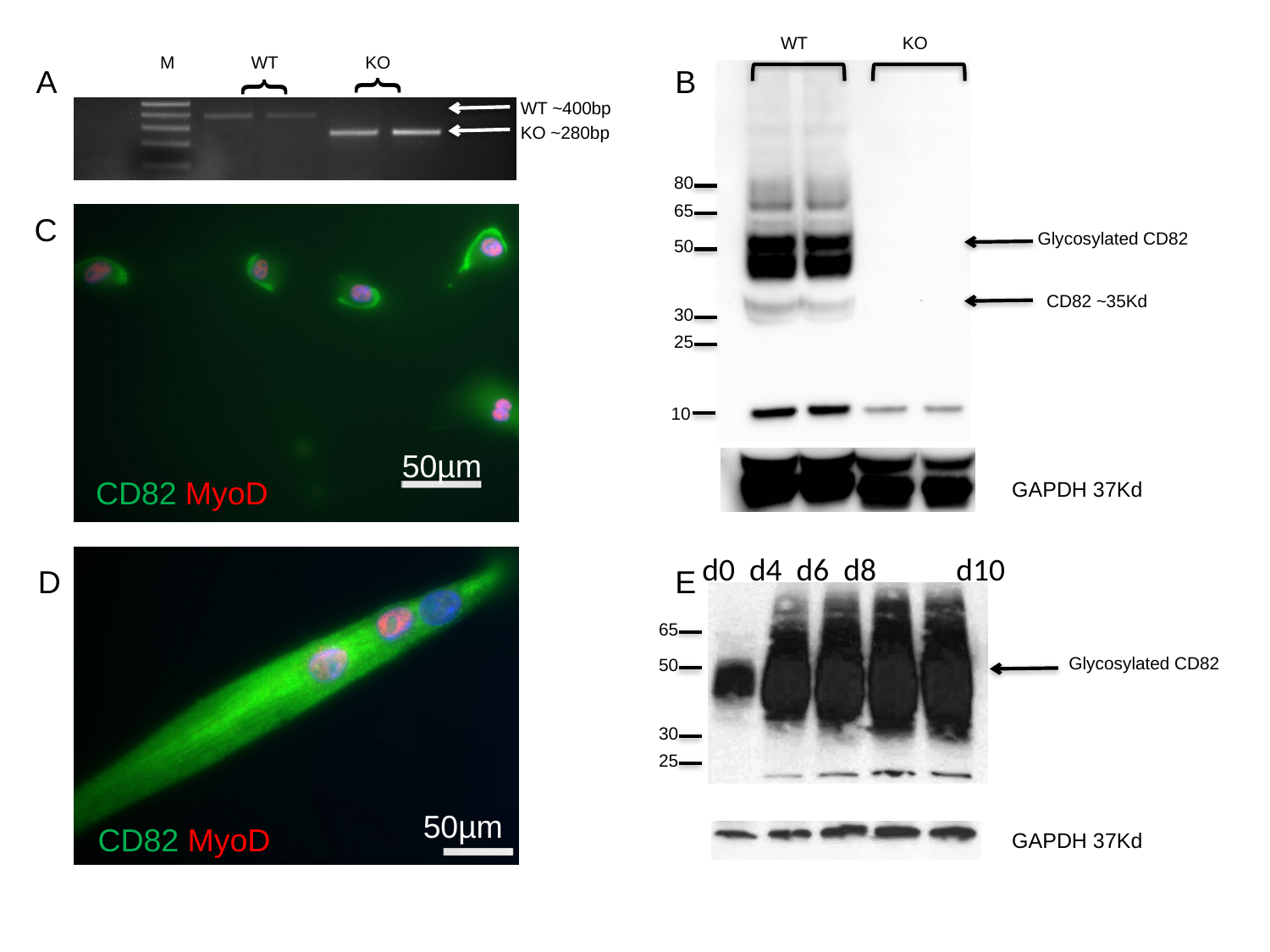

WT
KO
80
65
Glycosylated CD82
50
CD82 ~35Kd
30
25
10
GAPDH 37Kd
65
Glycosylated CD82
50
30
25
GAPDH 37Kd
M
WT
KO
{
{
A
B
WT ~400bp
KO ~280bp
C
50µm
CD82 MyoD
d0 d4 d6 d8 	d10
D
E
50µm
CD82 MyoD
