# Supplementary material for: Tetraspanin CD82 is necessary for muscle stem cell activation and supports dystrophic muscle function
Source: Skelet Muscle. 2020 Nov 27;10:34. doi: 10.1186/s13395-020-00252-3 (PMC7693590; doi:10.1186/s13395-020-00252-3)
Supplement: Supplementary file 6 — Additional file 6: Supplementary Table 1. List of primary antibodies used in this study. [file 13395_2020_252_MOESM6_ESM.pdf]

Supplementary Table 1. List of primary antibodies used in this study.

Antibody/name	Dilution	Item no.	Company	Application
Desmin	1:2000	Ab8592	AbCam	Western blot
CD82 (M35)	1:5000	ENH036	Kerafast	Western blot
CD82	1:100	Ab66400	AbCam	IF
PTEN	1:1000	9188	Cell Signaling	Western blot
pAKT(Ser473)	1:1000	4060	Cell Signaling	Western blot
AKT (total)	1:1000	2920	Cell Signaling	Western blot
mTOR2448	1:1000	5536	Cell Signaling	Western blot
mTOR (total)	1:1000	2972	Cell Signaling	Western blot
p-p70S6K	1:1000	9204	Cell Signaling	Western blot
p70S6K	1:1000	2708	Cell Signaling	Western blot
p4EBP1	1:1000	2855	Cell Signaling	Western blot
4EBP1 (total)	1:1000	9644	Cell Signaling	Western blot
MyoD	1:100	Sc-760 (M318)	Santa Cruz	IF
MyoD	1:100	G1 (sc377460)	Santa Cruz	IF
Ki67	1:500	9129S	CST	IF
H2A.X (S139)	1:400	2577S	CST	IF
Pax7	1:200	Concentrated supernatant	University of Iowa DSHB	IF
MF 20	1:50	Concentrated supernatant	University of Iowa DSHB	IF
